# Association between sexual violence and multiple high-risk fertility behaviours among women of reproductive age in sub-Saharan Africa

**DOI:** 10.1186/s12889-023-17444-3

**Published:** 2024-02-12

**Authors:** Richard Gyan Aboagye, Irene Esi Donkoh, Joshua Okyere, Abdul-Aziz Seidu, Bright Opoku Ahinkorah, Sanni Yaya

**Affiliations:** 1 Department of Family and Community Health, Fred N. Binka School of Public Health, University of Health and Allied Sciences, Hohoe, Ghana; 2 Department of Medical Laboratory Science, University of Cape Coast, Cape Coast, Ghana; 3 Department of Population and Health, University of Cape Coast, Cape Coast, Ghana; 4 Department of Nursing, College of Health Sciences, Kwame Nkrumah University of Science and Technology, Kumasi, Ghana; 5 Centre for Gender and Advocacy, Takoradi Technical University, Takoradi, Ghana; 6 College of Public Health, Medical and Veterinary Sciences, James Cook University, Townsville, Australia; 7 School of Clinical Medicine, University of New South Wales Sydney, Sydney, Australia; 8 School of International Development and Global Studies, University of Ottawa, Ottawa, Canada; 9 The George Institute for Global Health, Imperial College London, London, UK; 10 School of International Development and Global Studies, Faculty of Social Sciences, University of Ottawa, 120 University Private, Ottawa, ON K1N 6N5 Canada

**Keywords:** Sex offence, Violence, Abuse, sub-Saharan Africa, Women

## Abstract

**Background:**

Intimate partner violence has adverse outcomes on the sexual and reproductive health of women. In this study, we examined the association between sexual violence and multiple high-risk fertility behaviours (MHRFB) among women in sub-Saharan Africa (SSA).

**Methods:**

We conducted a cross-sectional analysis of data pooled from the most recent Demographic and Health Surveys of 20 countries in SSA. We included countries with most recent datasets conducted from 2015 to 2021 and had data on all variables included in the study. A weighted sample of 88,011 was included in the study. We used a multilevel binary logistic regression to examine the association between sexual violence and MHRFB, controlling for other covariates. The regression results were presented using adjusted odds ratio (aOR) with 95% confidence interval (CI). Statistical significance was set at *p* < 0.05.

**Results:**

The overall prevalence of MHRFB was 22.53% (95% CI: 22.26–22.81), which ranged from 9.94% in South Africa to 30.38% in Chad. For sexual violence, the pooled prevalence was 7.02% (95% CI: 6.86–7.19). Burundi (20.58%) and the Gambia (2.88%) reported the highest and lowest proportions, respectively. Women who experienced sexual violence were more likely to engage in MHRFB compared to those who did not experience sexual violence [aOR = 1.11, 95% CI: 1.02, 1.21].

**Conclusion:**

There is a positive association between sexual violence and the risk of MHRFB. Our findings underscore a need for sub-Saharan African countries to strengthen their efforts to reduce the occurrence of sexual violence in intimate partner relationships. To augment efforts and accelerate social change, sub-Saharan African countries can introduce pro-poor policies and interventions to improve the wealth status of women. Also, empowering women through the encouragement of attaining higher education would be a useful step in lowering the risk of MHRFB in SSA.

## Background

A common problem in the field of health care is intimate partner violence (IPV), characterized as the physical, emotional or sexual assault of a spouse or other intimate partner or both [1]. An estimated one in four women experience IPV, which threatens global public health [2]. However, geographical variations exist [3]. Evidence from the World Health Organization indicates that IPV rates range from 37% in least developed countries to 16–23% and 18–21% in Europe and Central, Eastern and South-Eastern Asia respectively. The rate in sub-Saharan Africa is 33% [4].

IPV has adverse health outcomes in about 3–13% of women globally which is implicated in post-traumatic stress disorder and mental disease [5]. Beyond this, the association between sexual violence and high-risk fertility behaviors has gained attention due to its potential implications for maternal and child health. High-risk fertility behaviour describes a situation where a woman experiences too-early or too-late age at birth, shorter birth interval, and a higher parity [6]. When a woman experiences two or more of the aforementioned situations, then that person is described as having experienced multiple high-risk fertility behavior (MHRFB). Evidence suggests that women who experienced abuse from an intimate partner have a 51% chance of becoming pregnant and are 41% more likely to do so within 18 months of the index pregnancy [7]. 

Literature shows that a plethora of individual and contextual factors contribute to women’s experience of high-risk fertility behavior. For instance, Das et al. [8] reported that increasing age and rural residency are significant risk factors for MHRFB. Another study has demonstrated that IPV exacerbates shorter birth interval, which is a component of MHRFB [9]. Similarly, Seidu et al. [10] found that being in a polygamous relationship exacerbated the risk of MHRFB in SSA. Also, studies have identified poverty, substance use, marital status, post-traumatic stress disorder, and conventional gender role belief as factors associated with MHRFB [11–13].

Beyond these established factors associated with MHRFB, there is a growing interest in how sexual violence influences MHRFB. It must first be noted that both sexual violence and MHRFB are two phenomena that are deeply rooted in socio-cultural norms and expectations. There is evidence [14, 15] suggesting that sexual violence facilitates the likelihood of early pregnancy – thus fulfilling a component of MHRFB. Similarly, a study conducted in Uganda and Zimbabwe [16] indicates that experience of sexual violence increases women’s likelihood of having shorter birth intervals. To the best of our knowledge, Das et al.’s study [8] is the only research that has comprehensively examined the association between sexual violence and all components of MHRFB. The result from their study [8] shows that MHRFB was 32% higher among women who had experienced sexual violence compared to those who had no such experience. While Das et al.’s study [8] provides a comprehensive appreciation of the association between sexual violence and MHRFB, it was conducted in Nepal which is outside of SSA. As indicated earlier, both sexual violence and MHRFB are rooted in socio-cultural norms. Therefore, the findings of Das et al. [8] may not be wholly applicable in the sub-Saharan African region. After extensive literature search, we found no currently published study that has examined the association between sexual violence and MHRFB in SSA. This is an important knowledge gap given the fact that SSA records high cases of early pregnancy, early sexual debut, shorter birth interval and high parity [17, 18]. Therefore, this study aims to narrow the knowledge gap by examining the association between sexual violence and MHRFB in SSA.

## Methods

### Data source and study design

We conducted a cross-sectional analysis of data from the most recent Demographic and Health Survey (DHS) of 20 countries in SSA. We included countries with recent dataset conducted from 2015 to 2021 and had data on all variables included in the study. In each of the 20 countries, we extracted the data from the individual recode file. The dataset for each country included in the study can be accessed via https://dhsprogram.com/data/available-datasets.cfm. The DHS is a nationally representative survey usually conducted periodically in over 90 low- and middle-income countries [19]. A cross-sectional design was used for the DHS and respondents were sampled using a multistage cluster sampling technique. The initial stage involved selecting sample points (clusters) consisting of enumeration areas (EAs). At the second stage, a systematic sampling technique was employed to sample the households from the EAs. All the women in selected households who met the inclusion criteria were included in the survey and were interviewed. Detailed sampling methodology has been published in the literature [20]. The survey collects data on several health indicators and social issues such as domestic violence and fertility using a structured questionnaire [19, 21]. In this study, we included 88,011 women in sexual unions: married or cohabiting in our final analysis (Table 1). We based on the Strengthening the Reporting of Observational Studies in Epidemiology (STROBE) checklist in drafting and reporting this paper [22].

**Table 1:** Description of study sample per country

Country	Year of survey	Weighted sample	Weighted percentage
1. Angola	2015–16	3,848	4.4
2. Benin	2017-18	4,318	4.9
3. Burundi	2016–17	4,840	5.5
4. Cameroon	2018	4,185	4.8
5. Ethiopia	2016	4,565	5.2
6. Gambia	2019–20	3,119	3.5
7. Liberia	2019–20	1,960	2.2
8. Madagascar	2021	5,125	5.8
9. Mali	2018	2,941	3.4
10. Mauritania	2019–21	4,387	5.0
11. Malawi	2015–16	6,670	7.6
12. Nigeria	2018	11,509	13.0
13. Rwanda	2019–20	4,052	4.6
14. Sierra Leone	2019	4,172	4.7
15. Chad	2014–15	4,775	5.4
16. Tanzania	2015–16	3,637	4.1
17. Uganda	2016	4,968	5.6
18. South Africa	2016	2,439	2.8
19. Zambia	2018	3,734	4.3
20. Zimbabwe	2015	2,767	3.1
**All countries**	**2015–2021**	**88,011**	**100.0**

### Variables

#### Outcome variable

MHRFB was the outcome variable. To derive this variable, four parameters were used. These parameters were mothers aged < 18 years at the time of delivery, mothers aged > 34 years at the time of delivery, short birth interval (< 24 months), and high parity (> 3 children) [23–27]. Any woman with at least two parameters was categorised as having MHRFB and was coded as 1 = yes whereas those with one or none were coded as 0 = no [8, 28].

#### Key explanatory variable

Sexual violence was the key explanatory variable. It was derived from three questions in the modified version of the conflict tactics scale of the domestic violence model [29]. The respondents were asked whether their partners ever physically forced them into unwanted sex; whether the partner ever forced them into other unwanted sexual acts; and whether the respondents have been physically forced to perform sexual acts which they did not want to. The response options in each of the 3 questions were “yes” and “no”. The categorisation used in this study was informed by literature [30, 31]. Women who responded ‘yes’ to at least one of the 3 questions were said to have experienced sexual violence and their responses were coded as ‘1 = yes’ whilst the responses of those who had never experienced any of the violent acts were categorised as ‘0 = no’.

### Covariates

We included 14 variables as covariates in the study. The covariates were grouped into individual and contextual level variables. The individual level variables consisted of age of the women, educational level, marital status, current working status, exposure to reading newspaper or magazine, exposure to listening to radio, exposure to watching television, age at first sex, age at first union, and contraceptive use. Sex of household head, wealth index, place of residence, and geographical sub-regions were the contextual level covariates. The sub-regions were drived based on the country’s location on the African continent. Table 3 describes the categories of the covariates included in the study. We based the selection of the covariates in the study on their availability in the DHS dataset and from the literature [8, 10].

### Statistical analyses

**Fig. 1:**
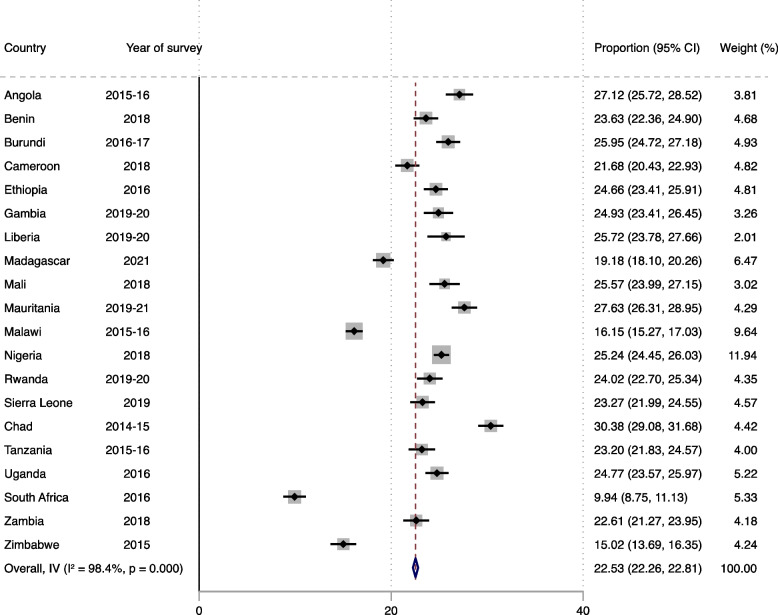
Prevalence of multiple high-risk fertility behaviour in sub-Saharan Africa

**Fig. 2:**
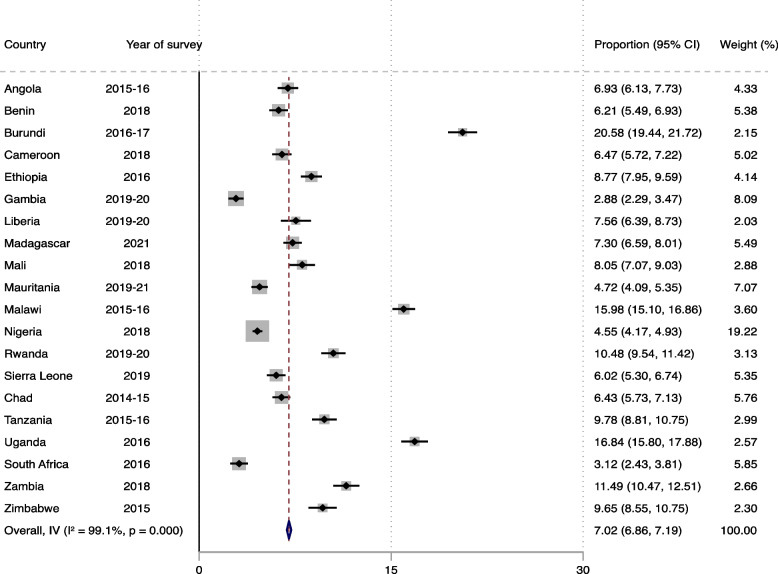
Prevalence of sexual violence in sub-Saharan Africa

We performed the statistical analysis in three stages using Stata software version 17.0 (Stata Corporation, College Station, TX, USA). At the initial stage, percentages with confidence intervals (CIs) were used to present the results of the prevalence of MHRFB and sexual violence (Figs. 1 and 2). The second stage was a bivariate analysis to determine the distribution of MHRFB across sexual violence, and the covariates as well as an estimated Pearson’s chi-square test of independence [*χ*^2^] at a *p*-value of less than 0.05 to show significant variables (Table 3). We used a multilevel binary logistic regression to examine the association between sexual violence and MHRFB, controlling for the covariates as the third stage. Model O showed the variance in MHRFB attributed to the clustering of the primary sampling units (PSUs). Model I was fitted to contain sexual violence and the individual level covariates. Model II included the contextual level covariates. The final model (Model III) was fitted to contain sexual violence and all the covariates. Stata command “melogit” was used in fitting the four models. Akaike’s Information Criterion (AIC) tests for model comparison was used in the analysis. We described the results in the final model for discussion since it is the model with the least AIC value and the highest log likelihood. All the results were presented using adjusted odds ratios (aOR) at 95% CI. All the analyses were weighted to correct for over and under-sampling, including the complex survey design to improve our findings’ generalizability.

### Ethical considerations

Ethical approval was not sought for this study. This is because the data is freely available in the public domain. However, prior to the commencement of the survey, ethical clearance was sought and all ethical guidelines governing the use of human subjects in the research were strictly adhered to. The detailed ethical guidelines are available at http://goo.gl/ny8T6X.

## Results

### Prevalence of multiple high‑risk fertility behaviours and sexual violence in sub-Saharan Africa

The overall prevalence of MHRFB was 22.53% (95% CI: 22.26–22.81) with Chad reporting the highest proportion (30.38%) while South Africa reported the lowest proportion (9.94%) (Fig. 1). Regarding sexual violence, we found the overall prevalence to be 7.02% (95% CI: 6.86–7.19). Burundi (20.58%) and the Gambia (2.88%) reported the highest and lowest proportions, respectively (Fig. 2).

### Prevalence of multiple high-risk fertility behaviours in sub-Saharan Africa

In Table 2, we present the individual distribution of the various components of MHRFB across the sub-Saharan African countries. Overall, 3.1% had their most recent birth before age 18 while 25.2% had their most recent birth after age 34. Regarding parity, 45.1% of the women were considered as having a high parity with Chad (59.8%) reporting the highest proportion. Similarly, 14.1% of the women had a short birth interval with Chad having the highest proportion (23.3%).

**Table 2:** Prevalence of multiple high-risk fertility behaviours

Country	Year of survey	Birth less than 18	Short birth interval	High parity	Age more than 34 years at birth
1. Angola	2015–16	4.6	18.4	49.2	24.3
2. Benin	2017-18	1.8	11.6	47.7	25.9
3. Burundi	2016–17	0.8	13.7	49.4	26.2
4. Cameroon	2018	4.2	16.9	44.5	21.7
5. Ethiopia	2016	2.2	15.5	50.4	24.9
6. Gambia	2019–20	1.8	10.6	48.3	31.7
7. Liberia	2019–20	2.8	11.0	47.0	27.2
8. Madagascar	2021	6.5	14.2	35.2	21.8
9. Mali	2018	4.1	16.3	51.5	26.0
10. Mauritania	2019–21	3.3	16.0	48.8	33.8
11. Malawi	2015–16	3.6	9.1	39.3	19.2
12. Nigeria	2018	2.6	16.7	47.3	26.7
13. Rwanda	2019–20	0.2	11.1	36.6	31.8
14. Sierra Leone	2019	3.2	11.8	45.4	25.0
15. Chad	2014–15	6.2	23.3	59.8	23.0
16. Tanzania	2015–16	2.6	12.8	43.1	26.9
17. Uganda	2016	3.1	17.8	49.2	23.0
18. South Africa	2016	2.0	6.0	17.2	23.7
19. Zambia	2018	3.3	10.7	46.1	22.7
20. Zimbabwe	2015	2.4	8.0	31.5	20.6
**All countries**	**2015–2021**	**3.1**	**14.1**	**45.1**	**25.2**

### Distribution of multiple high-risk fertility behaviours across sexual violence and the covariates

Table 3 shows the distribution of MHRFB across sexual violence and the covariates. There was no significant difference between MHRFB and sexual violence even though the experience of MHRFB was high among those who had not experienced sexual violence (23.4%). Women aged 45–59 years (69.1%), those with no formal education (33%), those married (24.3%), and those currently working (25%) had a higher percentage of MHRFB. These differences were significant. Also, women who had no exposure to reading newspaper (24.9%), no exposure to listening to the radio (25.1%), and no exposure to watching television (26%) reported higher proportions of MHRFB. The proportion of MHRFB was significantly higher among women who had their first union before age 15 (25.5%), as well as those who did not use contraceptives (24.4%), those from male-headed households (23.6%), and those from the the poorest wealth index households (27.6%). For the sub-region differences, the proportion of MHRFB was highest among women in Eastern Africa (24.1%) while those in Southern Africa reported the least proportion (9.9%).

**Table 3:** Bivariable analysis of multiple high-risk fertility behaviours across sexual violence and the covariates

Variable	Weighted sample	Weighted percentage	MHRFB	
			**Yes [95% CI]**	***P*-value**
**Sexual violence**				0.211
No	80,216	91.1	23.4 [23.0, 23.8]	
Yes	7,795	8.9	22.6 [21.5, 23.8]	
**Women’s age**				< 0.001
15–24	20,981	23.8	2.5 [2.3, 2.8]	
25–29	19,675	22.4	7.8 [7.4, 8.3]	
30–34	17,730	20.1	11.3 [10.6, 11.9]	
35–39	13,794	15.7	42.8 [41.7, 43.9]	
40–44	9,142	10.4	64.9 [63.6, 66.3]	
45–49	6,689	7.6	69.1 [67.6, 70.6]	
**Women’s educational level**				< 0.001
No education	30,567	34.7	33.0 [32.3, 33.7]	
Primary	31,307	35.6	23.0 [22.4, 23.7]	
Secondary	22,044	25.0	12.6 [12.0, 13.3]	
Higher	4,093	4.7	11.2 [9.9, 12.7]	
**Marital status**				< 0.001
Married	71,824	81.6	24.3 [23.9, 24.8]	
Cohabiting	16,187	18.4	18.8 [18.1, 19.6]	
**Current working status**				< 0.001
Not working	30,497	34.7	20.2 [19.5, 20.9]	
Working	57,514	65.3	25.0 [24.5, 25.5]	
**Exposed to reading newspaper**				< 0.001
No	73,952	84.0	24.9 [24.5, 25.4]	
Yes	14,059	16.0	14.9 [14.2, 15.7]	
**Exposed to listening to radio**				< 0.001
No	39,727	45.1	25.1 [24.5, 25.7]	
Yes	48,284	54.9	21.9 [21.4, 22.4]	
**Exposed to watching television**				< 0.001
No	54,724	62.2	26.0 [25.5, 26.5]	
Yes	33,287	37.8	18.9 [18.3, 19.5]	
**Age at first sex**				0.034
20 years and above	19,413	22.1	24.1 [23.2, 25.1]	
Below 20 years	68,598	77.9	23.1 [22.7, 23.5]	
**Age at first union**				< 0.001
Below 15 years	12,492	14.2	25.5 [24.4, 26.5]	
15–17 years	27,041	30.7	22.7 [22.0, 23.3]	
18 years and above	48,478	55.1	23.1 [22.6, 23.7]	
**Contraceptive use**				< 0.001
No	62,314	70.8	24.4 [23.9, 24.8]	
Yes	25,697	29.2	20.8 [20.2, 21.5]	
**Sex of household head**				< 0.001
Male	74,069	84.2	23.6 [23.2, 24.0]	
Female	13,942	15.8	21.8 [21.0, 22.7]	
**Wealth index**				< 0.001
Poorest	17,689	20.1	27.6 [26.7, 28.4]	
Poorer	18,073	20.5	25.5 [24.7, 26.4]	
Middle	17,780	20.2	25.3 [24.5, 26.1]	
Richer	17,578	20.0	21.9 [21.1, 22.8]	
Richest	16,891	19.2	15.9 [15.1, 16.7]	
**Place of residence**				< 0.001
Urban	29,707	33.8	18.6 [17.9, 19.3]	
Rural	58,304	66.2	25.7 [25.3, 26.2]	
**Sub-regions**				< 0.001
Southern	2,439	2.8	9.9 [8.4, 11.7]	
Central	11,670	13.2	23.9 [23.0, 24.9]	
Eastern	39,496	44.9	24.1 [23.5, 24.6]	
Western	34,406	39.1	23.2 [22.5, 24.0]	

### Association between sexual violence and multiple high-risk fertility behaviours women in sub-Saharan Africa

 Table 4 shows the results of the association between sexual violence and MHRFB. Women who experienced sexual violence [aOR = 1.11, 95% CI: 1.02, 1.21] were more likely to engage in MHRFB compared to those who did not experienced sexual violence. The likelihood of engaging in MHRFB increased with age with older women of reproductive age (45–49 years) [aOR = 125.8, 95% CI: 109.74, 144.14] reporting the highest odds compared to those aged 15–24. Higher odds of engaging in MHRFB was reported among women who used contraceptives [aOR = 1.11, 95% CI: 1.05, 1.19] and those residing in rural areas [aOR = 1.12, 95% CI: 1.04, 1.21] compared to those who did not use contraceptives or lived in urban areas.

**Table 4:** Association between multiple high-risk fertility behaviours and sexual violence among women in sub-Saharan Africa

Variable	Model O	Model I AOR [95% CI]	Model II AOR [95% CI]	Model III AOR [95% CI]
**Fixed effect model**				
**Sexual violence**				
No		1.00		1.00
Yes		1.14^**^ [1.05, 1.25]		1.11^*^ [1.02, 1.21]
**Women’s age**				
15–24		1.00		1.00
25–29		3.49^***^ [3.06, 3.97]		3.60^***^ [3.16, 4.11]
30–34		5.25^***^ [4.63, 5.95]		5.58^***^ [4.92, 6.34]
35–39		34.95^***^ [31.14, 39.23]		38.33^***^ [34.09, 43.10]
40–44		88.96^***^ [78.60, 100.68]		101.6^***^ [89.66, 115.20]
45–49		107.5^***^ [94.04, 122.85]		125.8^***^ [109.74, 144.14]
**Women’s educational level**				
No education		1.00		1.00
Primary		0.77^***^ [0.73, 0.82]		0.80^***^ [0.75, 0.85]
Secondary		0.46^***^ [0.42, 0.51]		0.59^***^ [0.54, 0.65]
Higher		0.29^***^ [0.24, 0.34]		0.42^***^ [0.35, 0.51]
**Marital status**				
Married		1.00		1.00
Cohabiting		1.00 [0.93, 1.07]		0.98 [0.91, 1.06]
**Current working status**				
Not working		1.00		1.00
Working		0.99 [0.93, 1.05]		0.92^**^ [0.86, 0.98]
**Exposed to listening to radio**				
No		1.00		1.00
Yes		0.93^*^ [0.88, 0.99]		0.96 [0.90, 1.02]
**Exposed to watching television**				
No		1.00		1.00
Yes		0.81^***^ [0.76, 0.86]		1.00 [0.93, 1.07]
**Exposed to reading newspaper**				
No		1.00		1.00
Yes		0.72^***^ [0.66, 0.79]		0.79^***^ [0.72, 0.87]
**Age at first sex**				
20 years and above		1.00		1.00
Below 20 years		1.07^*^ [1.00, 1.15]		1.02 [0.95, 1.09]
**Age at first union**				
Below 15 years		1.00		1.00
15–17 years		0.95 [0.87, 1.05]		0.95 [0.87, 1.04]
18 years and above		0.79^***^ [0.72, 0.86]		0.80^***^ [0.73, 0.87]
**Contraceptive use**				
No		1.00		1.00
Yes		1.06 [0.99, 1.12]		1.11^***^ [1.05, 1.19]
**Sex of household head**				
Male			1.00	1.00
Female			0.91^***^ [0.86, 0.96]	0.80^***^ [0.75, 0.86]
**Wealth index**				
Poorest			1.00	1.00
Poorer			0.90^***^ [0.84, 0.95]	0.87^***^ [0.80, 0.94]
Middle			0.90^**^ [0.85, 0.96]	0.83^***^ [0.76, 0.90]
Richer			0.77^***^ [0.72, 0.83]	0.69^***^ [0.63, 0.76]
Richest			0.55^***^ [0.51, 0.61]	0.49^***^ [0.44, 0.55]
**Place of residence**				
Urban			1.00	1.00
Rural			1.20^***^ [1.13, 1.28]	1.12^**^ [1.04, 1.21]
**Subregions**				
Southern			1.00	1.00
Central			2.59^***^ [2.12, 3.17]	5.34^***^ [4.23, 6.74]
Eastern			2.49^***^ [2.05, 3.03]	4.36^***^ [3.47, 5.48]
Western			2.42^***^ [1.99, 2.95]	3.92^***^ [3.11, 4.93]
**Random effect model**				
PSU variance (95% CI)	0.406 [0.320, 0.516]	0.588 [0.473, 0.732]	0.368 [0.291, 0.466]	0.551 [0.441, 0.689]
ICC	0.110	0.152	0.101	0.143
Wald chi-square	Reference	12241.67 (< 0.001)	508.94 (< 0.001)	12,566.10 (< 0.001)
**Model fitness**				
Log-likelihood	-173971.44	-116926.66	-171933.35	-115356.12
AIC	347946.9	233893.3	343888.7	230770.2
N	88011	88011	88011	88011
Number of clusters	1395	1395	1395	1395

*aOR* Adjusted odds ratios, *CI* Confidence Interval

*1.00* Reference category, *PSU* Primary Sampling Unit, *ICC* Intra-Class Correlation Coefficient, *AIC* Akaike’s Information Criterion

^*^p < 0.05

^**^p < 0.01

^***^p < 0.001

Compared to women with no education, those with primary [aOR = 0.80, 95% CI: 0.75, 0.85], secondary [aOR = 0.59, 95% CI: 0.54, 0.65], and higher education [aOR = 0.42, 95% CI: 0.35, 0.51] had lower odds of engaging in MHRFB. Also, women who were currently working [aOR = 0.92, 95% CI: 0.86, 0.98] had lower likelihood of engaging in MHRFB compared to those who were not working. The probability of engaging in MHRFB was lower among women who were exposed to reading newspapers [aOR = 0.79, 95% CI: 0.72, 0.87] and those residing in female-headed households [aOR = 0.80, 95% CI: 0.75, 0.86].

Women living in the richest wealth indexed households were less likely to engage in MHRFB [aOR = 0.49, 95% CI: 0.44, 0.55] compared to those from the poorest wealth indexed households. Similarly, the odds of having MHRFB was lower among women who entered into sexual union after age 18 [aOR = 0.80, 95% CI: 0.73, 0.87]. Compared to women from Southern Africa, those from Central Africa [aOR = 5.34, 95% CI: 4.23, 6.74], Eastern Africa [aOR = 4.36, 95% CI: 3.47, 5.48], and Western Africa [aOR = 3.92, 95% CI: 3.11, 4.93] were more likely to engage in MHRFB.

## Discussion

Engaging in MHRFB has been reported to be associated with several adverse health outcomes [8, 28]. The present study examined the association between sexual violence and MHRFB in SSA. Our findings revealed that 22.53% of women in SSA were engaged in MHRFB. With the exception of South Africa that reported a lower prevalence of MHRFB (9.94%) relative to the findings of a study conducted in India (13%) [8], all the remaining countries included in the study recorded a higher proportion. However, there were significant sub-regional differences in the proportion of MHRFB in SSA, with those in Central, Eastern, and Western Africa being at significantly higher risk of engaging in MHRFB compared to those in Southern Africa. Evidence from the study showed that age, contraceptive use, educational level, wealth index, household headship, place of residence, and media exposure were the covariates associated with MHRFB.

Our study revealed that women who experienced sexual violence were more likely to engage in MHRFB. The result is corroborated by Das et al.’s [8] study that reported a higher likelihood of MHRFB among women who experience sexual violence compared to those who did not. From our perspective, women who experienced sexual violence may not want to be in a situation where the power balance does not favor them. Hence, engaging in MHRFB may provide a sense of control or power over their bodies. We further posit that those who experience sexual violence may view engagement in MHRFB as a conduit to temporarily escape or get distracted from the negative emotions associated with the experience. Moreover, the increased likelihood of MHRFB could be attributed to unintended pregnancies, limited access to, and use of contraception, a product of sexual violence. For instance, sexual violence can increase the occurrence of unplanned pregnancies that could have contributed to short birth intervals or high parity both of which are parameters of MHRFB. In addition, unprotected sexual behavior—such as not using a condom—is a common practice among women who experience sexual violence. This behavior can lead to unintended pregnancies and shorter intervals between live deliveries [7, 8].

Our study also revealed that older women of reproductive age were at the highest risk of engaging in MHRFB. Similar findings have been reported in other low-and middle-income countries, including India [8] and Bangladesh [24]. We posit that the high risk of engaging in MHRFB among older women could be a result of the low-risk perception as well as other cultural factors. For instance, the cultural system in most sub-Saharan African countries expects older women of reproductive age to have more children [32]. This expectation can easily encourage older women of reproductive age to have shorter birth intervals and high parity, which are all constituents of MHRFB. Relatedly, the study showed that the probability of engaging in MHRFB was significantly lower among women who entered sexual unions after the age of 18. This is in contrast to Das et al.’s [8] study that found the likelihood of MHRFB to be higher among those who entered a sexual union after age 18.

Previous studies [8, 9, 27] have shown that contraceptive use is significantly associated with lower odds of engaging in MHRFB. This has been based on the argument that contraceptives and family planning aims to ensure planned birth intervals. Contrary to this body of knowledge, our study showed that women who used contraceptives were more likely to engage in MHRFB. Perhaps the counterintuitive result in this study can be explained from the perspective that while contraceptives are effective at ensuring an optimal birth interval, and to some extent reducing parity by limiting the likelihood of mistimed and unwanted pregnancies, it does not in any way affect whether women give birth early (before age 18), or late (i.e., after age 34).

Compared to urban-dwelling women, the risk of engaging in MHRFB was significantly higher among those residing in rural areas. This result concurs with the findings of other studies conducted in India [8] and Ethiopia [26]. Unlike urban-dwelling women who have easy access to health information regarding MHRFB, those in rural areas often lack access to this information [33], hence, making them more vulnerable to MHRFB. In addition, rural-dwelling women may be more likely than their counterparts in urban areas to conform to cultural norms and expectations that support high parity, shorter birth intervals, and early childbearing. Moreover, the hustle, work demands, and stress in urban areas may not offer residents the chance to have high parity and shorter birth intervals.

Consistent with the findings of previous studies [8, 26], we found a negative association between education and the risk of engaging in MHRFB. That is, the higher the level of education, the lower the likelihood of a woman engaging in MHRFB. Highly educated women tend to be well informed about the dangers of engaging in MHRFB. Hence, their decision not to engage in MHRFB [8]. Furthermore, educated women are more likely to better synthesize and understand the health information that they receive to make the best decisions regarding their health and wellbeing. Relatedly, we found the odds of MHRFB to be significantly lower among women who read the newspaper. Thus, suggesting a strong association between media exposure and the likelihood of engaging in MHRFB.

Also, the study showed that wealth index and current work status have a significant negative association with MHRFB. This implies that women in the richest wealth index and those who were currently working were less likely to engage in MHRFB compared to those in the poorest wealth index and among those not working, respectively. This is analogous to the finding of a previous study [8] that has found a strongly significant association between work status, wealth index, and MHRFB. The result is, however, in contrast to a study by Aragaw et al. [26] who found no significant association between wealth status and high-risk fertility behavior. We suggest that women of high wealth status may have more access to health information and knowledge about MHRFB, and, therefore, be able to make an informed decision. Women who were currently working and those with high wealth status may be economically empowered to negotiate for safer sex, thereby reducing the risk of unwanted and mistimed pregnancies that often contribute to shorter birth intervals and high parity [34]. This is further evident in one of our findings which showed that women in female-headed households were less likely to engage in MHRFB.

### Strengths and limitations

The major strength of this study lies in the use of a nationally representative DHS dataset to examine the association between sexual violence and MHRFB in 20 sub-Saharan African countries. Also, our study to the best of our knowledge is the first to examine the association between sexual violence and MHRFB in SSA. However, there are some inherent limitations. First, we are unable to establish a causal pathway between sexual violence and MHRFB since the study relied on cross-sectional data. While we established a strong association between sexual violence and MHRFB, it is difficult to show whether sexual violence occurred before MHRFB, or in the reverse. Also, the self-reported nature of the data could create grounds for recall and social desirability bias.

## Conclusion

Our study showed a positive association between sexual violence and the risk of MHRFB. Our findings underscore a need for sub-Saharan African countries to strengthen their efforts to reduce the occurrence of sexual violence in intimate partner relationships. To augment efforts and accelerate social change, sub-Saharan African countries can introduce pro-poor policies and interventions to improve the wealth status of women. By so doing, women would be empowered to better comprehend health information, and make informed decisions regarding their fertility behaviors. Also, encouraging to attain higher education would be a practical step towards reducing the risk of MHRFB among women in SSA.

## Data Availability

Data used in this study were obtained from the DHS Program and available at: https://dhsprogram.com/data/available-datasets.cfm.
